# Second Law Analysis of Unsteady MHD Viscous Flow over a Horizontal Stretching Sheet Heated Non-Uniformly in the Presence of Ohmic Heating: Utilization of Gear-Generalized Differential Quadrature Method

**DOI:** 10.3390/e21030240

**Published:** 2019-03-02

**Authors:** Muhammad Qasim, Muhammad Idrees Afridi, Abderrahim Wakif, T. Nguyen Thoi, Abid Hussanan

**Affiliations:** 1 Department of Mathematics, COMSATS University Islamabad (CUI) Park Road, Tarlai Kalan, Islamabad 455000, Pakistan; 2 Laboratory of Mechanics, Faculty of Sciences Aïn Chock, Hassan II University, B.P. 5366, Mâarif, Casablanca 20000, Morocco; 3 Division of Computational Mathematics and Engineering, Institute for Computational Science, Ton Duc Thang University, Ho Chi Minh City 700000, Vietnam; 4 Faculty of Civil Engineering Ton Duc Thang University, Ho Chi Minh City 700000, Vietnam; 5 Faculty of Mathematics and Statistics, Ton Duc Thang University, Ho Chi Minh City 700000, Vietnam

**Keywords:** entropy generation, unsteady flow, Bejan number, energy dissipation, Ohmic heating, Gear-Generalized Differential Quadrature Method

## Abstract

In this article, the entropy generation characteristics of a laminar unsteady MHD boundary layer flow are analysed numerically for an incompressible, electrically conducting and dissipative fluid. The Ohmic heating and energy dissipation effects are added to the energy equation. The modelled dimensional transport equations are altered into dimensionless self-similar partial differential equations (PDEs) through suitable transformations. The reduced momentum and energy equations are then worked out numerically by employing a new hybrid method called the Gear-Generalized Differential Quadrature Method (GGDQM). The obtained numerical results are incorporated in the calculation of the Bejan number and dimensionless entropy generation. Quantities of physical interest, like velocity, temperature, shear stress and heat transfer rate, are illustrated graphically as well as in tabular form. Impacts of involved parameters are examined and discussed thoroughly in this investigation. Exact and GGDQM solutions are compared for special cases of initial unsteady flow and final steady state flow. Furthermore, a good harmony is observed between the results of GGDQM and those given previously by the Spectral Relaxation Method (SRM), Spectral Quasilinearization Method (SQLM) and Spectral Perturbation Method (SPM).

## 1. Introduction

Physically, entropy is an assessment of molecular chaos or its randomness. As a thermally dynamic system becomes more disordered, the locations of the molecules become more and more uncertain and therefore their positions become less predictable and the entropy increases. The boost in the disorder of a thermodynamic system is termed entropy generation/production. Entropy generation determines the level of irreversibilities that accumulate during a process. With the increasing rate of entropy creation in any heated system, the quality of energy is reduced, that is, it destroys useful work and therefore reduces the thermal effectiveness of the system. Determining the range of entropy generation within the fluid flow area may help to improve system efficiency and achieve the optimal thermal or mechanical design [1,2,3,4,5,6,7,8,9,10].

Due to the vital applications of boundary layer flows in industries, technology, and manufacturing, many researchers have analysed boundary layer flows by applying different physical situations, such as magnetic field, heat source/sink, porous medium, suction/injection, combined mass and heat transfer, different velocity, and thermal boundary conditions. In particular, the interest in the boundary layer flow induced by a stretching sheet with heat transfer has developed rapidly in recent decades because of its many applications in numerous manufacturing and industrial processes, for example in the extrusion of polymer leaf from dye, the cooling mechanism of materials, fiberglass, paper manufacture, illustration of plastic films, casting process, etc. The innovative work on this topic is due to Crane [11], who originated the study of boundary layer flow past a stretching surface. Subsequent to this well-known work, several researchers have done a lot of research on this subject. There is a vast literature on flow over stretching surfaces, but we only mention a few very recent studies [12,13,14,15,16,17,18,19,20,21]. Moreover, most of the research done in this direction is for the steady flows. Only a few studies are available on the unsteady stream over a stretching area. Pop and Na [22] analyzed the unsteady flow over a stretching sheet. They obtained the analytical resolution of the dimensionless momentum equation by using a regular perturbation method. Chang et al. [23] discussed the unsteady flow of a viscous fluid over an impulsively stretching sheet. They obtained the perturbation series solution of self-similar boundary layer for small times and asymptotic analysis is also performed in order to obtain the solution for large times. Time-dependent boundary layer flow over a stretching sheet in a rotating fluid is examined by Nazar et al. [24]. They computed the numerical explanation of the problem by the Keller box method. Zheng et al. [25] reported the unsteady flow with combined effects of mass and heat transmission over an oscillatory stretching surface. Aurangzaib et al. [26] used the Keller box method to study the unsteady run of micropolar fluid above a vertical plate fixed in a permeable media. Malvandi et al. [27] investigated the heat source/sink impacts on the unsteady flow of a nanofluid over a permeable stretching sheet. Motsa and Makukula [28] studied the heat and mass transfer analysis of boundary layer flow of rotating fluid over a stretching sheet. Motsa [29] applied spectral homotopy analysis and local linearization method to solve self-similar equations of unsteady boundary layer flow induced by an impulsive stretching sheet. Vajravelu et al. [30] performed the combined heat and mass transfer analysis of unsteady flow past a shrinking surface in the presence of viscous dissipation and thermal radiation.

Lorentz force is generated when an electrically conducting fluid flow in the presence of magnetic field. The momentum equation is modified by adding the body force J×B per unit volume. Electrically conducting fluids have substantial applications in MHD accelerators, power generation, cooling filaments, and electrostatic filters. Due to substantial practical applications in industry, Andersson [31] analyzed electrically conducting fluids under the effects of a magnetic field. Three-dimensional unsteady MHD flow has been investigated by Xu et al. [32]. Recently, Sheikholeslami [33] reported the effects of Lorentz force and porous medium on entropy generation in a nanofluid. The effects of magnetic field on the flow of $Al_{2}O_{3}$-water nanofluid in a porous cavity were studied by Sheikholeslami [34] using the control volume finite element method.

Entropy generation analysis of boundary layer flows induced by a stretching sheet has been performed by various researchers [35,36,37,38,39,40,41,42,43,44]. However, no attention has been given to understanding the entropy generation in an unsteady boundary layer flow during linear stretching with Ohmic heating. Therefore, the present study concentrates on the heat transfer and entropy analyses of a magnetohydrodynamic unsteady flow of dissipative fluid with the existence of Lorentz force. The modelled nonlinear equations are solved numerically by utilizing an auto-adaptative implicit algorithm based on the Generalized Differential Quadrature Method (GDQM) [45,46,47] or the Gear Method (GM) [48] and discretizing the physical space domain into non-uniformly distributed grid points, which are generated simultaneously along with GDQM by the help of Gauss‒Lobatto collocation points [49,50,51,52]. Moreover, the present numerical results are portrayed and discussed thoroughly via various graphical and tabular illustrations, in order to examine the influences of several emerging key parameters, such as Prandtl number Pr, magnetic parameter M, Eckert number Ec, as well as the reduced dimensionless time ξ and the temperature difference parameter Ω.

The innovation of the current study lies essentially in the use of a new hybrid numerical method called Gear-Generalized Differential Quadrature Method (GGDQM) for studying thermodynamically the present unsteady boundary layer flow problem in the presence of viscous dissipation and Ohmic heating. The robustness and efficiency of the numerical results given by GGDQM are also compared analytically and numerically by considering the existing published results and introducing the notion of CPU time.

## 2. Flow and Heat Transfer Analysis

**Figure 1:**
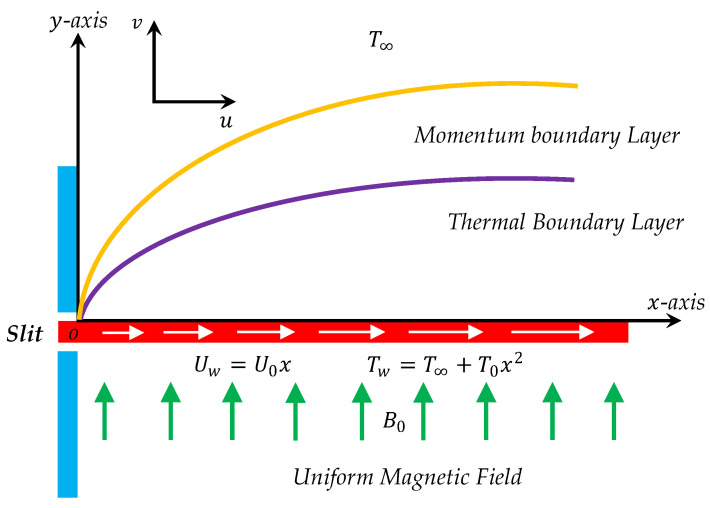
Flow configuration with Cartesian coordinate system.

As schematically portrayed in Figure 1, we consider a two-dimensional unsteady laminar forced convective flow of an incompressible, viscous, and electrically conducting fluid driven by a linearly stretching horizontal surface, in the presence of a vertical applied magnetic field of constant strength $B_{o},$ in such a way that the induced magnetic field is neglected under the assumption of a small magnetic Reynolds number. Initially (i.e., t=0), the studied fluid and the sheet surface y=0 are stationary and have a constant temperature $T_{\infty }$. After this time (i.e., t>0), the sheet surface y=0 is stretched linearly along the positive x-direction with a velocity $U_{w}=U_{o}x$ and heated non-uniformly by an imposed nonlinear thermal boundary condition of the form $T_{w}=T_{\infty }+T_{o}x^{2}$, where $U_{o}$ and $T_{o}$ are two dimensional constants that characterize the present unsteady boundary layer flow.

In the presence of an external magnetic field, viscous dissipation and Joule heating effects, the continuity, momentum, and energy equations governing the present unsteady boundary layer flow are written as follows

(1) $$\frac{\partial u}{\partial x}+\frac{\partial v}{\partial y}=0$$

(2) $$\rho \left(\frac{\partial u}{\partial t}+u\frac{\partial u}{\partial x}+v\frac{\partial u}{\partial y}\right)=\mu \frac{\partial^{2}u}{\partial y^{2}}-\sigma B_{o}^{2}u,$$

(3) $$\left(\rho C_{p}\right)\left(\frac{\partial T}{\partial t}+u\frac{\partial T}{\partial x}+v\frac{\partial T}{\partial y}\right)=k\frac{\partial^{2}T}{\partial y^{2}}+\mu {\left(\frac{\partial u}{\partial y}\right)}^{2}+\sigma B_{o}^{2}u^{2},$$

with the following appropriate boundary conditions

(4) $$u=U_{w}=U_{o}x,\;v=0,\;T_{w}=T_{\infty }+T_{o}x^{2}\;\mathrm{at}\;y=0,$$

(5) $$u\to 0,\;T\to T_{\infty }\;\mathrm{as}\;y\to \infty ,$$

where t is the dimensional time, (u,v) are the tangential and normal fluid velocity components, respectively, μ is the dynamic viscosity of the incompressible fluid, ρ is the density, σ is the electrical conductivity of the conducting fluid, $B_{o}$ is the uniform magnetic field applied vertically along the y-direction, T is the temperature of the fluid throughout the boundary layer, k is the thermal conductivity of the working fluid and $\left(\rho C_{p}\right)$ is the fluid heat capacitance.

By introducing the following dimensionless quantities

(6) $$\eta ={\left(\frac{U_{o}}{\upsilon \xi }\right)}^{1/2}y,\;u=U_{o}xf_{\eta }\left(\xi ,\eta \right),\;v=-{\left(U_{o}\upsilon \xi \right)}^{1/2}f\left(\xi ,\eta \right),\;\theta \left(\xi ,\eta \right)=\frac{T-T_{\infty }}{T_{w}-T_{\infty }}.$$

Equations (1)–(5) reduce to

(7) $$\frac{\partial f_{\eta }}{\partial \xi_{}}=\frac{f_{\eta \eta \eta }}{\left(1-\xi \right)\xi }+\frac{\eta_{}f_{\eta \eta }}{2\xi }-\frac{M^{2}f_{\eta }}{\left(1-\xi \right)}+\frac{ff_{\eta \eta }}{\left(1-\xi \right)}-\frac{f_{\eta }^{2}}{\left(1-\xi \right)},$$

(8) $$\frac{\partial \theta }{\partial \xi }=\frac{\theta_{\eta \eta }}{\mathrm{Pr}\xi \left(1-\xi \right)}+\frac{\eta_{}\theta_{\eta }}{2\xi }+\frac{f\theta_{\eta }}{\left(1-\xi \right)}-\frac{2_{}f_{\eta \;}\theta }{\left(1-\xi \right)}+\frac{Ec_{}f_{\eta \eta }^{2}}{\xi \left(1-\xi \right)}+\frac{Ec_{}M^{2}f_{\eta }^{2}}{\left(1-\xi \right)},$$

(9) $$f\left(\xi ,\eta \right)=0,\;f_{\eta }\left(\xi ,\eta \right)=1,\;\theta \left(\xi ,\eta \right)=1\;\mathrm{at}\;\eta =0\;\mathrm{for}\;0\leq \xi \leq 1$$

(10) $$\theta \left(\xi ,\eta \right)=0,\;f_{\eta }\left(\xi ,\eta \right)=0\;\mathrm{as}\;\eta \to \infty \;\mathrm{for}\;0\leq \xi \leq 1.$$

Here, the subscripts η, ηη and ηηη used above for f and θ designate the first, second and third partial derivatives, respectively, with respect to the variable η.

The continuity equation described above by Equation (1) is satisfied identically by introducing the stream function ψ(t,x,y), such that

(11) $$\left\{ \begin{array}{l} \left(u,v\right)=\left(\frac{\partial \psi }{\partial y},-\frac{\partial \psi }{\partial x}\right),\\ \psi ={\left(U_{o}\upsilon \xi \right)}^{1/2}xf\left(\xi ,\eta \right). \end{array}\right.$$

Note that in the case of a non-uniform wall temperature heating condition with linear spatial variation $\left(i.e.,\;T_{w}=T_{\infty }+T_{o}x\right)$, the dimensionless energy equation takes the following form

(12) $$\frac{\partial \theta }{\partial \xi }=\frac{\theta_{\eta \eta }}{\mathrm{Pr}\xi \left(1-\xi \right)}+\frac{\eta_{}\theta_{\eta }}{2\xi }+\frac{f\theta_{\eta }}{\left(1-\xi \right)}-\frac{f_{\eta \;}\theta }{\left(1-\xi \right)}+\frac{Ec_{}f_{\eta \eta }^{2}}{\xi \left(1-\xi \right)}+\frac{Ec_{}M^{2}f_{\eta }^{2}}{\left(1-\xi \right)}.$$

In addition, the non-dimensional physical parameters ξ, Pr, Ec and M appearing above in Equations (7) and (8) are defined mathematically as

(13) $$\xi =1-e^{-\tau }\;(\mathrm{reduced}\;\mathrm{dimensionless}\;\mathrm{time}),\;\mathrm{Pr}=\frac{\nu \left(\rho C_{p}\right)}{k}\;(\mathrm{Prandtl}\;\mathrm{number}),\;Ec=\frac{U_{w}^{2}}{C_{p}\left(T_{w}-T_{\infty }\right)}\;(\mathrm{Eckert}\;\mathrm{number}),\;M^{2}=\frac{\sigma B_{o}^{2}}{\rho U_{w}}\;(\mathrm{magnetic}\;\mathrm{parameter}).$$

Here, τ is the dimensionless time and ν is the kinematic viscosity of fluid, where $\tau =U_{o}t$ and ν=μ/ρ.

For the present boundary layer flow problem, the skin friction coefficient $Cf_{x}$ and the local Nusselt number $Nu_{x}$ are defined as

(14) $$C_{fx}=-\frac{\mu }{\rho U_{w}^{2}}{\frac{\partial u}{\partial y}|}_{{}_{y=0}},$$

(15) $$Nu_{x}=-{\frac{x}{\left(T_{w}-T_{\infty }\right)}\frac{\partial T}{\partial y}|}_{{}_{y=0}}.$$

In dimensionless form, these engineering quantities reduce to

(16) Rex1/2Cfx=−ξ−1/2fηη(ξ,0),

(17) Rex−1/2Nux=−ξ−1/2θη(ξ,0).

### 2.1. Closed Form Solutions

Generally, for a non-conservative fluidic system, the viscous dissipation and Joule heating terms cannot be neglected in the energy equation (i.e., Ec≠0). Therefore, the closed-form analytical solutions for f(ξ,η) and θ(ξ,η) cannot be obtained for the present dynamical system. On the contrary, the exact solutions of the dimensionless stream function f(ξ,η) for the limiting cases of initial unsteady flow (i.e., ξ=0) and final steady state flow (i.e., ξ=1) can be found easily from Equation (7). Hence, by making use of the boundary conditions related to f(ξ,η), the special solutions for f(0,η) and f(1,η) are expressed formally as follows

(18) $$f\left(0,\eta \right)=\eta_{}erfc\left(\frac{\eta }{2}\right)+\frac{2}{\sqrt{\pi }}\left[1-exp\left(-\frac{\eta^{2}}{4\;}\right)\right],$$

(19) $$f\left(1,\eta \right)=\frac{1-\exp\left[-{\left(M^{2}+1\right)}^{1/2}\eta \right]}{{\left(M^{2}+1\right)}^{1/2}}.$$

By virtue of these solution expressions and the boundary conditions of f(ξ,η) and θ(ξ,η), we obtain from Equation (8) the following results

(20) $$\left\{ \begin{array}{l} f_{\eta \eta }(0,0)=-\frac{1}{\sqrt{\pi }},\\ \theta_{\eta \eta }(0,0)=-\frac{\mathrm{Pr}Ec}{\pi }, \end{array}\right.$$

(21) $$\left\{ \begin{array}{l} f_{\eta \eta }(1,0)=-{\left(M^{2}+1\right)}^{1/2},\\ \theta_{\eta \eta }(1,0)=\mathrm{Pr}\left(2-Ec\left(2M^{2}+1\right)\right). \end{array}\right.$$

The validity of the numerical results presented in this paper is confirmed in the next section by comparing our findings with those developed analytically for the physical quantities $f_{\eta \eta }\left(0,0\right)$, $f_{\eta \eta }\left(1,0\right)$, $\theta_{\eta \eta }\left(0,0\right)$ and $\theta_{\eta \eta }\left(1,0\right)$, as shown in Equations (20) and (21).

### 2.2. Second Law Analysis

As is well known, the volumetric entropy production rate of an electrically conducting fluid flowing in the presence of an externally applied magnetic field is defined thermodynamically by

(22) $${{\dot{S}}^{\prime \prime \prime }}_{gen}=k{\left(\frac{\nabla T}{T}\right)}^{2}+\left(\frac{\;\mu }{T}\right)\Psi +\left(\frac{1}{\sigma T}\right)J^{2}.$$

Here, ∇T refers to the temperature gradient vector, Ψ denotes the viscous dissipation function of the incompressible Newtonian fluid and J represents the current density vector, such that

(23) $$\left\{ \begin{array}{l} \Psi =2{\left(\frac{\partial u}{\partial x}\right)}^{2}+2{\left(\frac{\partial v}{\partial y}\right)}^{2}+{\left(\frac{\partial u}{\partial y}+\frac{\partial v}{\partial x}\right)}^{2},\\ J=\sigma \left(V\times B_{0}\right). \end{array}\right.$$

Using the boundary layer approximations, Equation (22) reduce to

(24) $${\dot{S}}_{gen}^{\prime \prime \prime }=\frac{k\;}{T^{2}}{\left(\frac{\partial T}{\partial y}\right)}^{2}+\frac{\mu \;}{T}{\left(\frac{\partial u}{\partial y}\right)}^{2}+\frac{\sigma B_{o}^{2}u^{2}}{T}.$$

As shown in Equation (24), the expression of ${\dot{S}}_{gen}^{\prime \prime \prime }$ depicts three bases of entropy production. These thermodynamic sources are the heat transfer, the viscous friction and the Ohmic heating, respectively.

The dimensionless form of Equation (24) is called entropy generation number Ns. This thermodynamic quantity is given by

(25) $$Ns=\frac{{\dot{S}}_{gen}^{\prime \prime \prime }}{{\left({\dot{S}}_{gen}^{\prime \prime \prime }\right)}_{o}},$$

where ${\left({\dot{S}}_{gen}^{\prime \prime \prime }\right)}_{o}$ represents a characteristic entropy generation of the studied system.

By employing the similarity transformations shown in Equation (6), we get

(26) $$Ns=\frac{\theta_{\eta }^{2}}{{\left(\theta +\Omega \right)}^{2}}+\frac{\mathrm{Pr}Ec_{}f_{\eta \eta }^{2}}{\left(\theta +\Omega \right)}+\frac{M^{2}Ec\mathrm{Pr}\xi_{}f_{\eta }^{2}}{\left(\theta +\Omega \right)}.$$

Here, Ω denotes the temperature difference parameter, where

(27) $${\left({\dot{S}}_{gen}^{\prime \prime \prime }\right)}_{o}=\frac{k\left(\rho C_{p}\right)}{\mu_{}\xi },$$

(28) $$\Omega =\frac{T_{w}-T_{\infty }}{T_{\infty }}.$$

Another interesting thermodynamic quantity called the Bejan number Be can be computed from the different entropic terms shown in Equation (26) as follows

(29) $$Be=\frac{N_{h}}{N_{h}+N_{f}+N_{m}},$$

where

(30) $$\left\{ \begin{array}{l} N_{h}=\frac{\theta_{\eta }^{2}}{{\left(\theta +\Omega \right)}^{2}},\\ N_{f}=\frac{\mathrm{Pr}Ec_{}f_{\eta \eta }^{2}}{\left(\theta +\Omega \right)},\\ N_{m}=\frac{M^{2}Ec\mathrm{Pr}\xi_{}f_{\eta }^{2}}{\left(\theta +\Omega \right)}. \end{array}\right.$$

After introducing the expressions of $N_{h}$, $N_{f}$ and $N_{m}$ into Equation (29), we get

(31) $$Be=\frac{\theta_{\eta }^{2}}{\theta_{\eta }^{2}+\mathrm{Pr}Ec\left(\theta +\Omega \right)\left(f_{\eta \eta }^{2}+M^{2}\xi_{}f_{\eta }^{2}\right)}.$$

From the definition of the Bejan number Be, it is obvious that Be is always comprised between 0 and 1. The zero value of Be implies that the combined contribution of fluid friction and magnetic field completely overrides the heat transfer effect, while the unit value of Be indicates that the heat transfer mechanism is the only cause of entropy creation. On the contrary, it is found that the heat transfer and the combined effects of magnetic field and viscous dissipation can make an equal entropic contribution in cases where Be=0.5.

From the implementation point of view, the unsteady boundary layer flow problem under consideration can be further simplified by considering the following changes

(32) $$\{\begin{array}{l} \eta =\eta_{\infty }\chi ,\\ f\left(\xi ,n\right)=f\left(\xi ,n_{\infty }\chi \right)=F\left(\xi ,\chi \right),\\ \theta \left(\zeta ,n\right)=\theta \left(\xi ,n_{\infty }\chi \right)=\Theta \left(\xi ,\chi \right). \end{array}$$

It is worth noting that the dimensionless space variable χ is introduced above instead of η for reducing the physical space domain from [0,∞] to [0,1], in which $\eta_{\infty }$ represents the optimum value of the boundary layer thickness.

Keeping in mind the above transformations, Equations (7) and (8) with their corresponding boundary conditions (i.e., Equations (9) and (10)) reduce to

(33) $$\frac{\partial F_{\chi }}{\partial \xi_{}}=\frac{F_{\chi \chi \chi }}{\eta_{\infty }^{2}\left(1-\xi \right)\xi }+\frac{\chi_{}F_{\chi \chi }}{2\xi }-\frac{M^{2}F_{\chi }}{\left(1-\xi \right)}+\frac{FF_{\chi \chi }}{\eta_{\infty }\left(1-\xi \right)}-\frac{F_{\chi }^{2}}{\eta_{\infty }\left(1-\xi \right)},$$

(34) $$\frac{\partial \Theta }{\partial \xi }=\frac{\Theta_{\chi \chi }}{\mathrm{Pr}\eta_{\infty }^{2}\xi \left(1-\xi \right)}+\frac{\chi_{}\Theta_{\chi }}{2\xi }+\frac{F_{}\Theta_{\chi }}{\eta_{\infty }\left(1-\xi \right)}-\frac{2F_{\chi \;}\Theta }{\eta_{\infty }\left(1-\xi \right)}+\frac{Ec_{}F_{\chi \chi }^{2}}{\eta_{\infty }^{4}\xi \left(1-\xi \right)}+\frac{Ec_{}M^{2}F_{\chi }^{2}}{\eta_{\infty }^{2}\left(1-\xi \right)},$$

(35) $$F\left(\xi ,\chi \right)=0,\;F_{\chi }\left(\xi ,\chi \right)=\eta_{\infty },\;\Theta \left(\xi ,\chi \right)=1\;\mathrm{at}\;\chi =0\;\mathrm{for}\;0\leq \xi \leq 1,$$

(36) $$F_{\chi }\left(\xi ,\chi \right)=0,\;\Theta \left(\xi ,\chi \right)=0\;\mathrm{as}\;\chi \to 1\;\mathrm{for}\;0\leq \xi \leq 1,$$

Also, the dimensionless physical quantities Rex1/2Cfx, Rex−1/2Nux, Ns and Be become

(37) Rex1/2Cfx=−ξ−1/2Fχχ(ξ,0)η∞2,

(38) Rex−1/2Nux=−ξ−1/2Θχ(ξ,0)η∞,

(39) $$Ns=\frac{\Theta_{\chi }^{2}}{\eta_{\infty }^{2}{\left(\Theta +\Omega \right)}^{2}}+\frac{\mathrm{Pr}Ec_{}F_{\chi \chi }^{2}}{\eta_{\infty }^{4}\left(\Theta +\Omega \right)}+\frac{M^{2}Ec\mathrm{Pr}\xi_{}F_{\chi }^{2}}{\eta_{\infty }^{2}\left(\Theta +\Omega \right)},$$

(40) $$Be=\frac{\eta_{\infty \;}^{2}\Theta_{\chi }^{2}}{\eta_{\infty }^{2}\Theta_{\chi }^{2}+\mathrm{Pr}Ec\left(\Theta +\Omega \right)\left(F_{\chi \chi }^{2}+\eta_{\infty \;}^{2}M^{2}\xi_{}F_{\chi }^{2}\right)}.$$

## 3. Solution Methodology

Due to the unsteadiness of the studied boundary layer flow problem and its nonlinear dynamical behaviour, the governing partial differential equations (PDEs) along with their associated boundary conditions (i.e., Equations (1)–(5)) are subjected to several necessary simplifications and suitable similarity transformations before being solved numerically by means of a powerful numerical tool. For this purpose, the resulting set of coupled nonlinear differential equations and boundary conditions (i.e., Equations (33)–(36)) is handled numerically using the Gear-Generalized Differential Quadrature Method (GGDQM), in order to reach a precision to the tenth decimal place as the standard of convergence (see Table 1).

### 3.1. Gear-Generalized Differential Quadrature Method (GGDQM)

For realizing a fine spatial discretization for the variable χ, it is more useful to use GDQM with the modified Gauss‒Lobatto grid points $\chi_{i}$, which are given by

(41) $$\chi_{i}=\frac{1}{2}-\frac{1}{2}cos\left(\frac{\pi i-\pi }{N-1}\right).$$

Here, *N* is the total number of Gauss-Lobatto collocation points, where 1 ≤ *i* ≤ *N*.

Accordingly, the functions *F* (*ξ*, *x*) and Θ (*ξ*, *x*) defined above in Equation (32) can be approximated at a collocation point $\chi =\chi_{i}$ by

(42) $$\left\{ \begin{array}{l} F^{\left(n\right)}\left(\xi ,\chi_{i}\right)=\sum_{j=1}^{N}d_{ij}^{\left(n\right)}\;F_{j}\left(\xi \right)\;\mathrm{for}\;1\leq i\leq N,\\ \Theta^{\left(n\right)}\left(\xi ,\chi_{i}\right)=\sum_{j=1}^{N}d_{ij}^{\left(n\right)}\;\Theta_{j}\left(\xi \right)\;\mathrm{for}\;1\leq i\leq N. \end{array}\right.$$

In addition, the weighting coefficients *d_ij_^(n)^* appearing in Equation (42) are given by Shu [45] as follows

(43) $$\left\{ \begin{array}{ll} d_{ij}^{\left(n\right)}=\frac{\overset{N}{\underset{k=1,\;k\neq i}{\prod }}\left(\chi_{i}-\chi_{k}\right)}{\left(\chi_{i}-\chi_{j}\right)\overset{N}{\underset{k=1,\;k\neq j}{\prod }}\left(\chi_{j}-\chi_{k}\right)} & \;\mathrm{for}\;i\neq j\;\mathrm{and}\;1\leq i,j\leq N,\;\mathrm{when}\;n=1,\\ d_{ij}^{\left(n\right)}=-\overset{N}{\underset{j=1,j\neq i}{\sum }}d_{ij}^{\left(1\right)}\; & \;\mathrm{for}\;i=j\;\mathrm{and}\;1\leq i,j\leq N,\;\mathrm{when}\;n=1,\;\\ d_{ij}^{\left(n\right)}=n\left[d_{ii}^{\left(n-1\right)}d_{ij}^{\left(1\right)}-\frac{d_{ij}^{\left(n-1\right)}}{\left(\chi_{i}-\chi_{j}\right)}\right] & \;\mathrm{for}\;i\neq j\;\mathrm{and}\;1\leq i,j\leq N,\;\mathrm{when}\;n\geq 2,\\ d_{ij}^{\left(n\right)}=-\overset{N}{\underset{j=1,j\neq i}{\sum }}d_{ij}^{\left(n\right)} & \;\mathrm{for}\;i=j\;\mathrm{and}\;1\leq i,j\leq N,\;\mathrm{when}\;n\geq 2. \end{array}\right.$$

Here, n represents the order of differentiation with respect to the variable χ.

After substituting the discretized form of F(ξ,χ) and Θ(ξ,χ) with their partial derivatives into Equations (33)–(36), we get the following semi-discrete system

(44) $$\left(S_{\xi }\right):\left\{ \begin{array}{lll} F_{1}\left(\xi \right)=0, & & \\ \sum_{j=1}^{N}d_{1j}^{\left(n\right)}\;F_{j}\left(\xi \right)-\eta_{\infty }=0,\\ \sum_{j=1}^{N}d_{ij}^{\left(1\right)}\;\frac{\partial F_{j}\left(\xi \right)}{\partial \xi }=L_{F_{{}_{\xi }}}+NL_{F_{{}_{\xi }}} & \;\mathrm{for}\;3\leq i\leq N-1,\\ \sum_{j=1}^{N}d_{Nj}^{\left(n\right)}\;F_{j}\left(\xi \right)=0, & \\ \Theta_{1}\left(\xi \right)-1=0, & \\ \frac{\partial \Theta_{i}\left(\xi \right)}{\partial \xi }=L_{\Theta_{{}_{\xi }}}+NL_{\Theta_{{}_{\xi }}} & \;\mathrm{for}\;2\leq i\leq N-1,\\ \Theta_{N}\left(\xi \right)=0, & \\ \mathrm{where}\;0<\xi <1. & \end{array}\right.$$

The linear and nonlinear parts $L_{F_{\xi }}$, $L_{\Theta_{\xi }}$, $NL_{F_{\xi }}$ and $NL_{\Theta_{\xi }}$ arising from Equations (33) and (34) are given by

(45) $$L_{F_{\xi }}=\frac{1}{\eta_{\infty }^{2}\left(1-\xi \right)\xi }\left(\sum_{j=1}^{N}d_{ij}^{\left(3\right)}\;F_{j}\left(\xi \right)\right)+\frac{\chi_{i}}{2\xi }\left(\sum_{j=1}^{N}d_{ij}^{\left(2\right)}\;F_{j}\left(\xi \right)\right)-\frac{M^{2}}{\left(1-\xi \right)}\left(\sum_{j=1}^{N}d_{ij}^{\left(1\right)}\;F_{j}\left(\xi \right)\right),$$

(46) $$L_{\Theta_{\xi }}=\frac{1}{\mathrm{Pr}\eta_{\infty }^{2}\xi \left(1-\xi \right)}\left(\sum_{j=1}^{N}d_{ij}^{\left(2\right)}\;\Theta_{j}\left(\xi \right)\right)+\frac{\chi_{i}}{2\xi }\left(\sum_{j=1}^{N}d_{ij}^{\left(1\right)}\;\Theta_{j}\left(\xi \right)\right),$$

(47) $$NL_{F_{\xi }}=\frac{1}{\eta_{\infty }\left(1-\xi \right)}F_{i}\left(\xi \right)\left(\sum_{j=1}^{N}d_{ij}^{\left(2\right)}\;F_{j}\left(\xi \right)\right)-\frac{1}{\eta_{\infty }\left(1-\xi \right)}{\left(\sum_{j=1}^{N}d_{ij}^{\left(1\right)}\;F_{j}\left(\xi \right)\right)}^{2},$$

(48) $$NL_{\Theta_{\xi }}=\left\{\begin{array}{l} \frac{1}{\eta_{\infty }\left(1-\xi \right)}\left[F_{i}\left(\xi \right)\left(\sum_{j=1}^{N}d_{ij}^{\left(1\right)}\;\Theta_{j}\left(\xi \right)\right)-2\left(\sum_{j=1}^{N}d_{ij}^{\left(1\right)}\;F_{j}\left(\xi \right)\right)\Theta_{i}\left(\xi \right)\right]\\ +\frac{Ec}{\left(1-\xi \right)}\left[\frac{1}{\eta_{\infty }^{4}\xi }{\left(\sum_{j=1}^{N}d_{ij}^{\left(2\right)}\;F_{j}\left(\xi \right)\right)}^{2}+\frac{M^{2}}{\eta_{\infty }^{2}}{\left(\sum_{j=1}^{N}d_{ij}^{\left(1\right)}\;F_{j}\left(\xi \right)\right)}^{2}\right] \end{array}\right\}.$$

It is worth pointing out that the solutions of the initial unsteady flow (i.e., ξ=0) and final steady state flow (i.e., ξ=1) can be found numerically by solving successively the following nonlinear algebraic systems

(49) $$\left(S_{0}\right):\left\{ \begin{array}{ll} F_{1}\left(0\right)=0, & \\ \sum_{j=1}^{N}d_{1j}^{\left(n\right)}\;F_{j}\left(0\right)-\eta_{\infty }=0, & \\ L_{F_{{}_{0}}}+NL_{F_{{}_{0}}}=0 & \;\mathrm{for}\;3\leq i\leq N-1,\\ \sum_{j=1}^{N}d_{Nj}^{\left(n\right)}\;F_{j}\left(0\right)=0, & \\ \Theta_{1}\left(0\right)-1=0, & \\ L_{\Theta_{{}_{0}}}+NL_{\Theta_{{}_{0}}}=0 & \;\mathrm{for}\;2\leq i\leq N-1,\\ \Theta_{N}\left(0\right)=0, & \\ \mathrm{where}\;\xi =0, & \end{array}\right.$$

(50) $$\left(S_{1}\right):\left\{ \begin{array}{ll} F_{1}\left(1\right)=0, & \\ \sum_{j=1}^{N}d_{1j}^{\left(n\right)}\;F_{j}\left(1\right)-\eta_{\infty }=0, & \\ L_{F_{{}_{1}}}+NL_{F_{{}_{1}}}=0 & \;\mathrm{for}\;3\leq i\leq N-1,\\ \sum_{j=1}^{N}d_{Nj}^{\left(n\right)}\;F_{j}\left(1\right)=0, & \\ \Theta_{1}\left(1\right)-1=0, & \\ L_{\Theta_{{}_{1}}}+NL_{\Theta_{{}_{1}}}=0 & \;\mathrm{for}\;2\leq i\leq N-1,\\ \Theta_{N}\left(1\right)=0, & \\ \mathrm{where}\;\xi =1. & \end{array}\right.$$

In the above limiting cases, the linear and nonlinear parts $\left(L_{F_{0}},L_{F_{1}}\right)$, $\left(L_{\Theta_{0}},L_{\Theta_{1}}\right)$, $\left(NL_{F_{0}},NL_{F_{1}}\right)$ and $\left(NL_{\Theta_{0}},NL_{\Theta_{1}}\right)$ shown above are expressed by

(51) $$\left\{ \begin{array}{l} L_{F_{0}}=\frac{1}{\eta_{\infty }^{2}}\left(\sum_{j=1}^{N}d_{ij}^{\left(3\right)}\;F_{j}\left(0\right)\right)+\frac{\chi_{i}}{2}\left(\sum_{j=1}^{N}d_{ij}^{\left(2\right)}\;F_{j}\left(0\right)\right),\\ L_{F_{1}}=\frac{1}{\eta_{\infty }^{2}}\left(\sum_{j=1}^{N}d_{ij}^{\left(3\right)}\;F_{j}\left(1\right)\right)-M^{2}\left(\sum_{j=1}^{N}d_{ij}^{\left(1\right)}\;F_{j}\left(1\right)\right), \end{array}\right.$$

(52) $$\left\{ \begin{array}{l} L_{\Theta_{0}}=\frac{1}{\mathrm{Pr}\eta_{\infty }^{2}}\left(\sum_{j=1}^{N}d_{ij}^{\left(2\right)}\;\Theta_{j}\left(0\right)\right)+\frac{\chi_{i}}{2}\left(\sum_{j=1}^{N}d_{ij}^{\left(1\right)}\;\Theta_{j}\left(0\right)\right),\\ L_{\Theta_{1}}=\frac{1}{\mathrm{Pr}\eta_{\infty }^{2}}\left(\sum_{j=1}^{N}d_{ij}^{\left(2\right)}\;\Theta_{j}\left(1\right)\right), \end{array}\right.$$

(53) $$\left\{ \begin{array}{l} NL_{F_{0}}=0,\\ NL_{F_{1}}=\frac{1}{\eta_{\infty }}F_{i}\left(1\right)\left(\sum_{j=1}^{N}d_{ij}^{\left(2\right)}\;F_{j}\left(1\right)\right)-\frac{1}{\eta_{\infty }}{\left(\sum_{j=1}^{N}d_{ij}^{\left(1\right)}\;F_{j}\left(1\right)\right)}^{2}, \end{array}\right.$$

(54) $$\left\{ \begin{array}{l} NL_{\Theta_{0}}=\frac{Ec}{\eta_{\infty }^{4}}{\left(\sum_{j=1}^{N}d_{ij}^{\left(2\right)}\;F_{j}\left(0\right)\right)}^{2},\\ NL_{\Theta_{1}}=\left[\begin{array}{l} \frac{1}{\eta_{\infty }}\left(F_{i}\left(\xi \right)\left(\sum_{j=1}^{N}d_{ij}^{\left(1\right)}\;\Theta_{j}\left(1\right)\right)-2\left(\sum_{j=1}^{N}d_{ij}^{\left(1\right)}\;F_{j}\left(1\right)\right)\Theta_{i}\left(1\right)\right)\\ +Ec\left(\frac{1}{\eta_{\infty }^{4}}{\left(\sum_{j=1}^{N}d_{ij}^{\left(2\right)}\;F_{j}\left(1\right)\right)}^{2}+\frac{M^{2}}{\eta_{\infty }^{2}}{\left(\sum_{j=1}^{N}d_{ij}^{\left(1\right)}\;F_{j}\left(1\right)\right)}^{2}\right) \end{array}\right]. \end{array}\right.$$

By utilizing the Newton-Raphson Method (NRM), the nonlinear algebraic systems $\left(S_{0}\right)$ and $\left(S_{1}\right)$ can be handled and then solved accurately, in order to find the numerical estimate values of the solutions $\left\{\left(F_{i}\left(0\right),\Theta_{i}\left(0\right)\right)\;\mathrm{and}\;\left(F_{i}\left(1\right),\Theta_{i}\left(1\right)\right)\;/\;1\leq i\leq N\right\}$. In this unsteady boundary layer flow problem, the solutions $\left\{\left(F_{i}\left(0\right),\Theta_{i}\left(0\right)\right)/\;1\leq i\leq N\right\}$ of the initial unsteady flow (i.e., ξ=0) corresponding to the algebraic system (*S_0_*) are taken as the initial conditions for the problem under consideration. Therefore, for generating numerically the general solutions $\left\{\left(F_{i}\left(\xi \right),\Theta_{i}\left(\xi \right)\right)\;/\;1\leq i\leq N\;\mathrm{and}\;0<\xi <1\right\}$ with $\eta_{\infty }=15$, the non-autonomous differential system *S_ξ_* along with the initial conditions $\left\{\left(F_{i}\left(0\right),\Theta_{i}\left(0\right)\right)/\;1\leq i\leq N\right\}$ is integrated temporarily using an auto-adaptative implicit algorithm based on the Gear Method (GM). Furthermore, to achieve an absolute accuracy of the order of $10^{-10}$, it is found that the dimensionless time-step size Δξ and the number of collocation points N must be selected as $\Delta \xi =10^{-5}$ and N=150 in all subsequent analyses.

Under the above convergence criterion, the dimensionless physical quantities of interest Rex1/2Cfx, Rex−1/2Nux, Ns and Be can then be computed numerically from the solutions $\left\{\left(F_{i}\left(\xi \right),\Theta_{i}\left(\xi \right)\right)\;/\;1\leq i\leq N\;\mathrm{and}\;0\leq \xi \leq 1\right\}$ as follows

(55) Rex1/2Cfx=−ξ−1/2(∑j=1Nd1j(2) Fj(ξ))η∞2,

(56) Rex−1/2Nux=−ξ−1/2(∑j=1Nd1j(1) Θj(ξ))η∞,

(57) $$Ns\left(\xi ,\chi_{i}\right)=\frac{1}{\eta_{\infty }^{2}{\left(\Theta_{i}+\Omega \right)}^{2}}{\left(\sum_{j=1}^{N}d_{ij}^{\left(1\right)}\;\Theta_{j}\left(\xi \right)\right)}^{2}+\left[\begin{array}{l} \frac{\mathrm{Pr}Ec_{}}{\eta_{\infty }^{4}\left(\Theta_{i}+\Omega \right)}{\left(\sum_{j=1}^{N}d_{ij}^{\left(2\right)}\;F_{j}\left(\xi \right)\right)}^{2}+\\ \frac{M^{2}Ec\mathrm{Pr}\xi_{}}{\eta_{\infty }^{2}\left(\Theta_{i}+\Omega \right)}{\left(\sum_{j=1}^{N}d_{ij}^{\left(1\right)}\;F_{j}\left(\xi \right)\right)}^{2} \end{array}\right],$$

(58) $$Be\left(\xi ,\chi_{i}\right)=\frac{\eta_{\infty }^{2}{\left(\sum_{j=1}^{N}d_{ij}^{\left(1\right)}\;\Theta_{j}\left(\xi \right)\right)}^{2}}{\eta_{\infty }^{2}{\left(\sum_{j=1}^{N}d_{ij}^{\left(1\right)}\;\Theta_{j}\left(\xi \right)\right)}^{2}+\mathrm{Pr}Ec\left(\Theta_{i}+\Omega \right)\left[\begin{array}{l} {\left(\sum_{j=1}^{N}d_{ij}^{\left(2\right)}\;F_{j}\left(\xi \right)\right)}^{2}+\\ \eta_{\infty \;}^{2}M^{2}\xi_{}{\left(\sum_{j=1}^{N}d_{ij}^{\left(1\right)}\;F_{j}\left(\xi \right)\right)}^{2} \end{array}\right]}.$$

Also, the discretized forms of the non-dimensional velocity $f_{\eta }\left(\xi ,\eta \right)$ and temperature θ(ξ,η) can be deduced as follows

(59) $$\left\{ \begin{array}{l} f_{\eta }(\xi ,\eta_{i})=\frac{1}{\eta_{\infty }}\left(\sum_{j=1}^{N}d_{ij}^{\left(1\right)}\;F_{j}\left(\xi \right)\right),\\ \theta (\xi ,\eta_{i})=\Theta_{i}, \end{array}\right.$$

where

(60) $$\eta_{i}=\frac{\eta_{\infty }}{2}-\frac{\eta_{\infty }}{2}cos\left(\frac{\pi i-\pi }{N-1}\right).$$

### 3.2. Validation of the Numerical Results

To authenticate the exactness of our solution methodology developed in this investigation by the Gear-Generalized Differential Quadrature Method (GGDQM), several numerical simulations are carried out using the Matlab software, in order to compare the numerical findings with our analytical solutions found for $f_{\eta \eta }\left(0,0\right)$, $f_{\eta \eta }\left(1,0\right)$, $\theta_{\eta \eta }\left(0,0\right)$ and $\theta_{\eta \eta }\left(1,0\right)$ (i.e., Equations (20) and (21)) and those obtained numerically by Motsa et al. [53] for $f_{\eta \eta }\left(\xi ,0\right)$ via the Spectral Relaxation Method (SRM) and the Spectral Quasilinearization Method (SQLM), as shown in Table 2, Table 3, Table 4 and Table 5. Also, GGDQM is further tested by computing the values of $\theta_{\eta \eta }\left(\xi ,0\right)$ from Equation (12) by considering the absence of viscous dissipation and Joule heating effects (i.e., Ec=0). These values are evaluated for different values of Pr and compared in Table 1 with those obtained by Agbaje and Motsa [54] with the help of the Spectral Perturbation Method (SPM) and the Spectral Relaxation Method (SRM). As expected, it is found from Table 1, Table 2, Table 3, Table 4 and Table 5 that there is a good agreement between the compared results. Hence, the accuracy of our GGDQM numerical code is strengthened by validating our findings against the analytical and numerical results of some limiting cases. Furthermore, the numerical results listed in Table 5 for CPU time prove that GGDQM is a fast implementation method compared with SRM and SQLM, where the time required for GGDQM to generate the results shown in Table 5 is less than 7 s.

## 4. Results and Discussion

**Figure 2:**
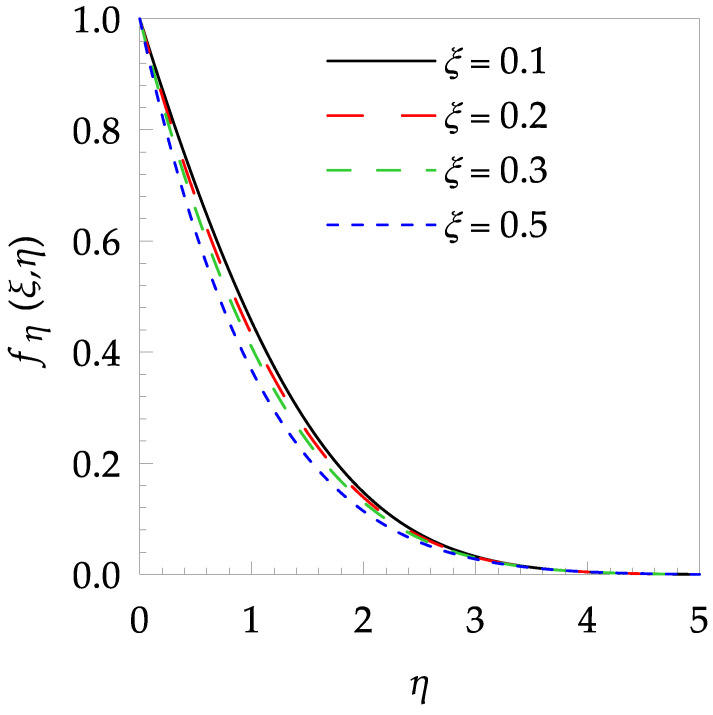
Velocity profile $f_{\eta }\left(\xi ,t\right)$ for some values of ξ, when Pr=6, M=0.8 and Ec=0.7.

**Figure 3:**
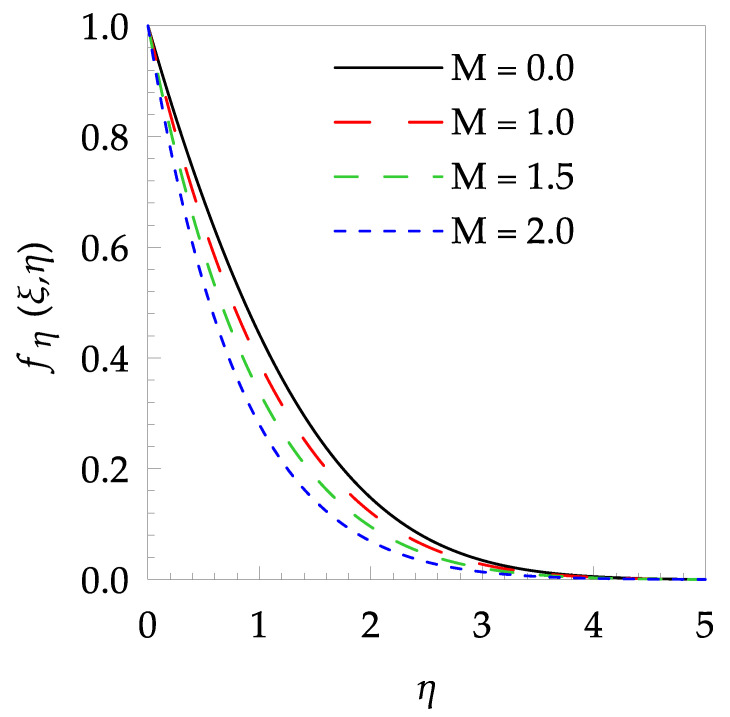
Velocity profile $f_{\eta }\left(\xi ,t\right)$ for some values of M, when ξ=0.3, Pr=5 and Ec=0.5.

**Figure 4:**
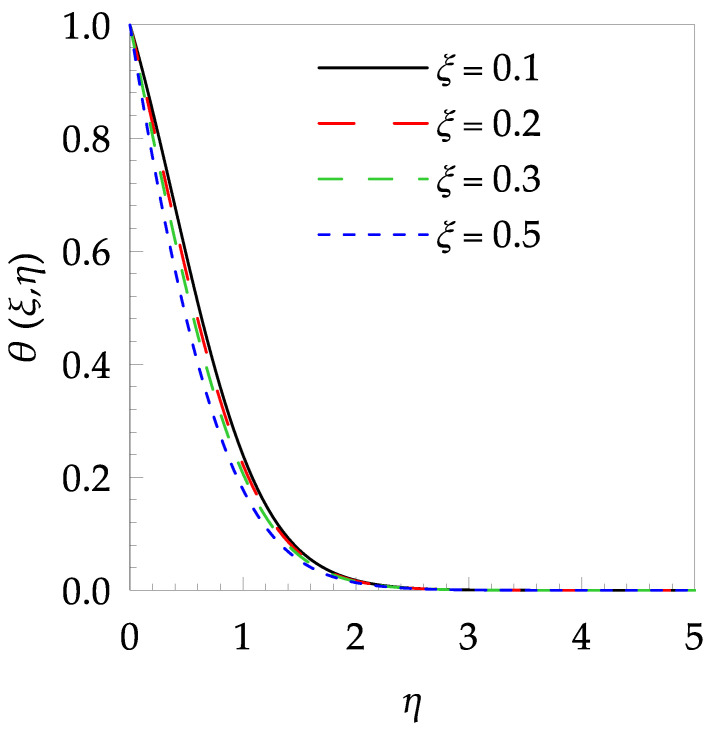
Temperature profile θ(ξ,t) for some values of ξ, when Pr=6.0, M=0.8 and Ec=0.7.

**Figure 5:**
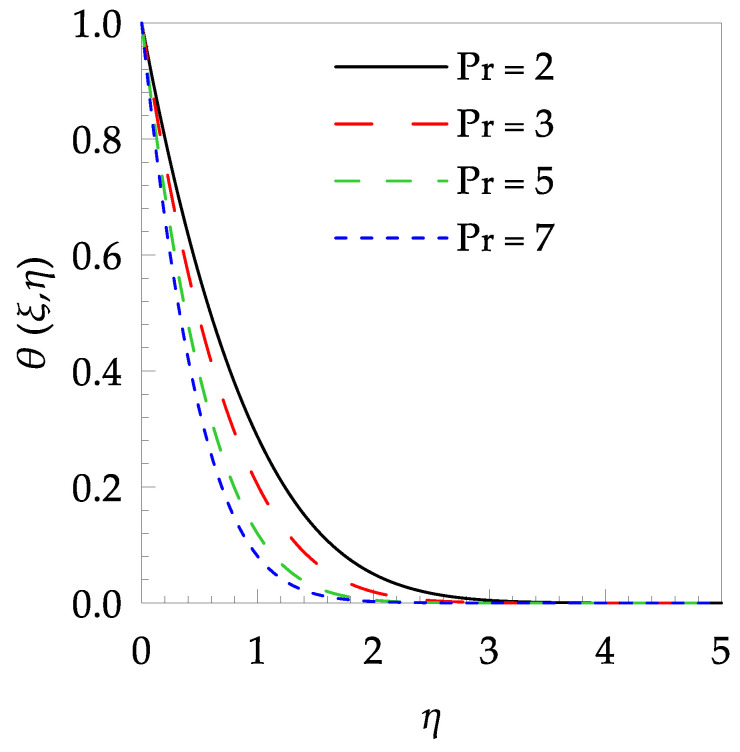
Temperature profile θ(ξ,t) for some values of Pr, when ξ=0.5, M=1.5 and Ec=0.2.

**Figure 6:**
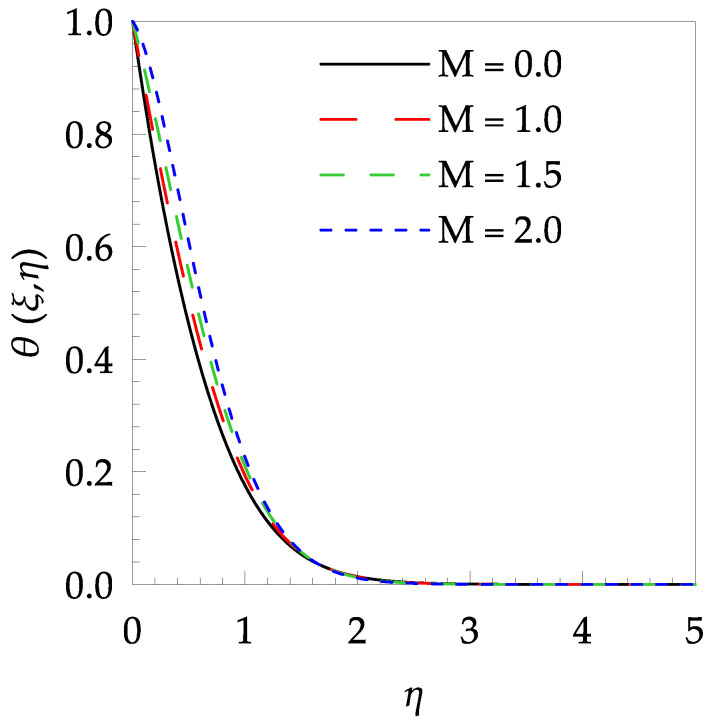
Temperature profile θ(ξ,t) for some values of M, when ξ=0.5, Pr=5 and Ec=0.5.

**Figure 7:**
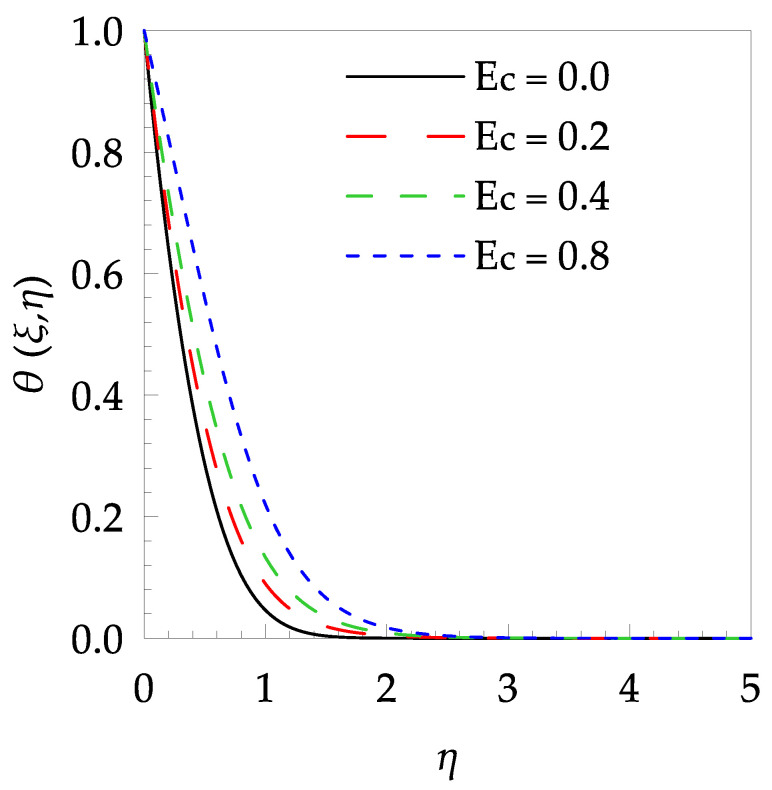
Temperature profile θ(ξ,t) for some values of Ec, when ξ=0.2, Pr=7 and M=0.5.

**Figure 8:**
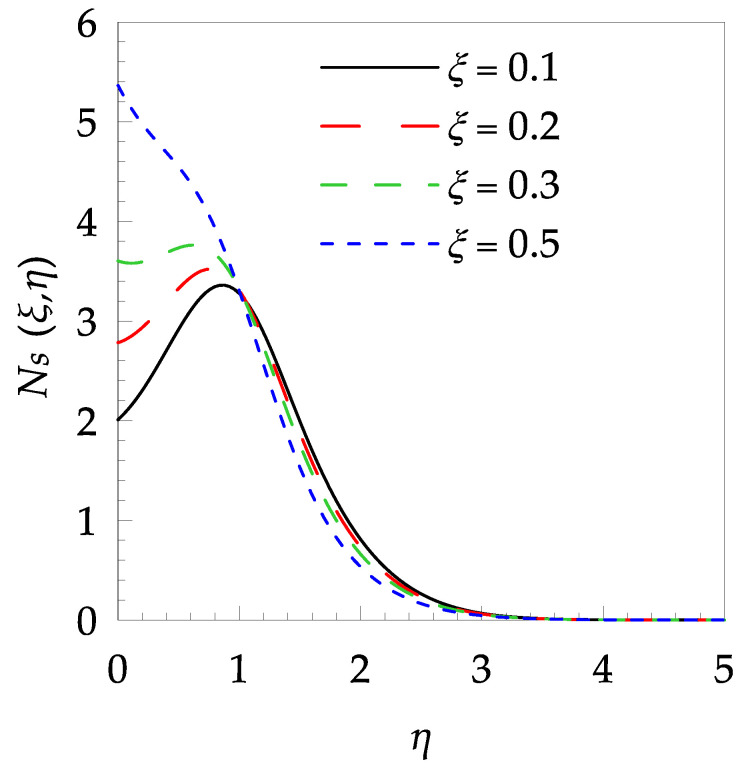
Entropy generation profile Ns for some values of ξ, when Pr=6, Ω=0.2, M=0.8 and Ec=0.7.

**Figure 9:**
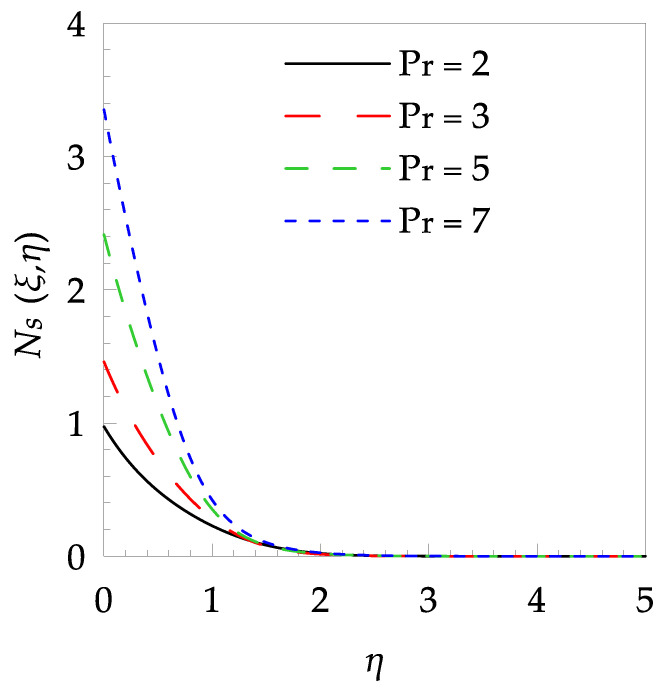
Entropy generation profile Ns for some values of Pr, when ξ=0.5, Ω=0.8, M=1.5 and Ec=0.2.

**Figure 10:**
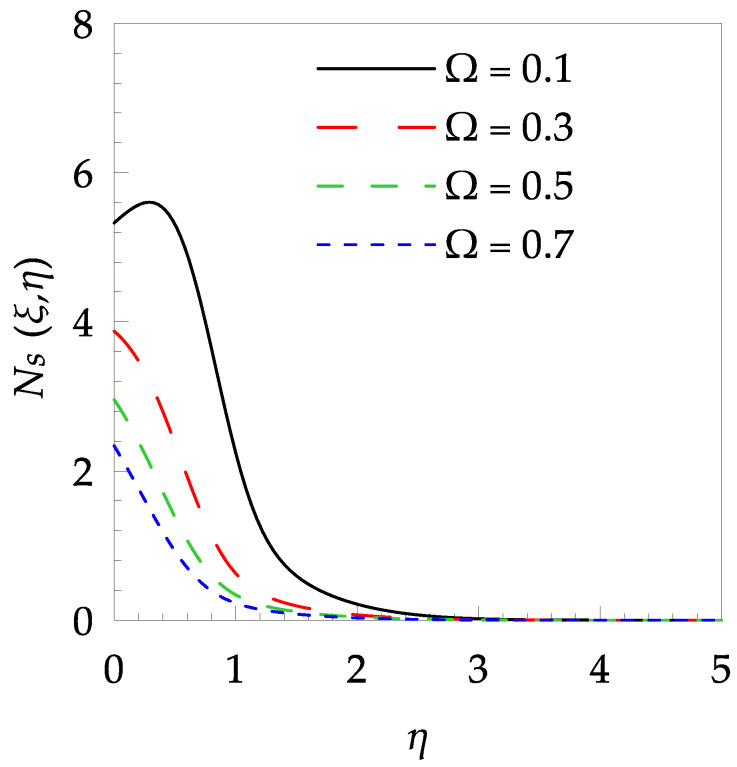
Entropy generation profile Ns for some values of Ω, when ξ=0.4, Pr=7, M=0.5 and Ec=0.1.

**Figure 11:**
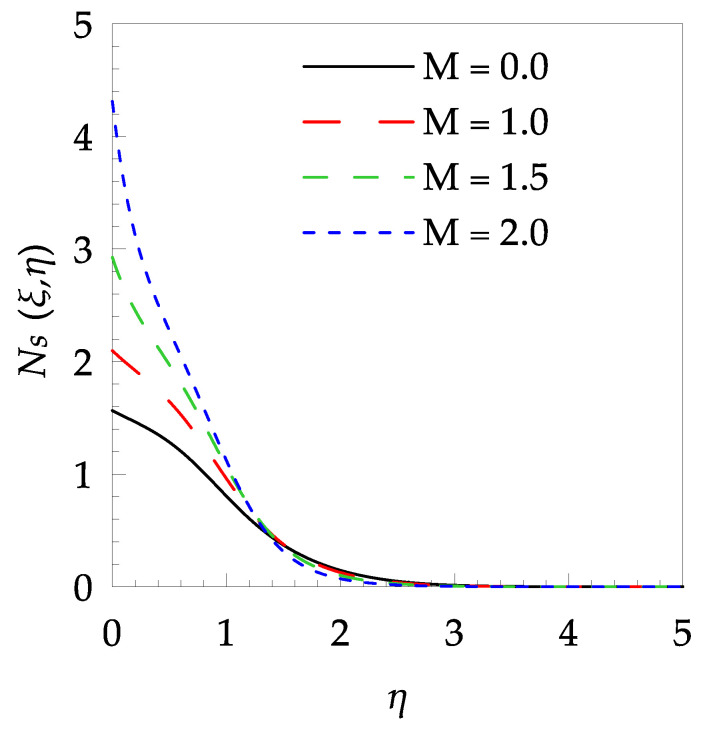
Entropy generation profile Ns for some values of M, when ξ=0.3, Pr=5, Ω=0.6 and Ec=0.5.

**Figure 12:**
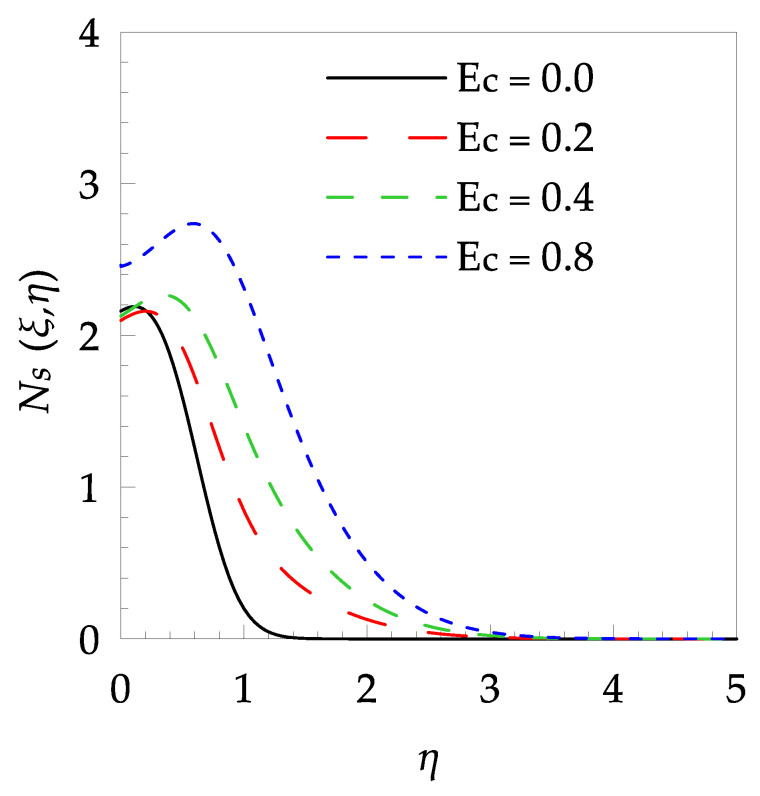
Entropy generation profile Ns for some values of Ec, when ξ=0.2, Pr=7, Ω=0.4 and M=0.5.

**Figure 13:**
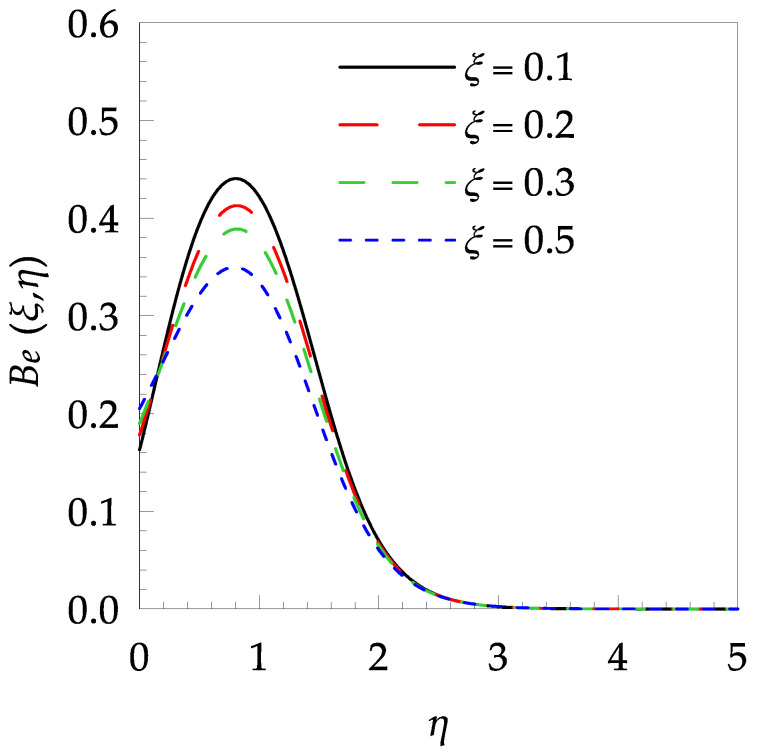
Bejan number profile Be for some values of ξ, when Pr=6, Ω=0.2, M=0.8 and Ec=0.7.

**Figure 14:**
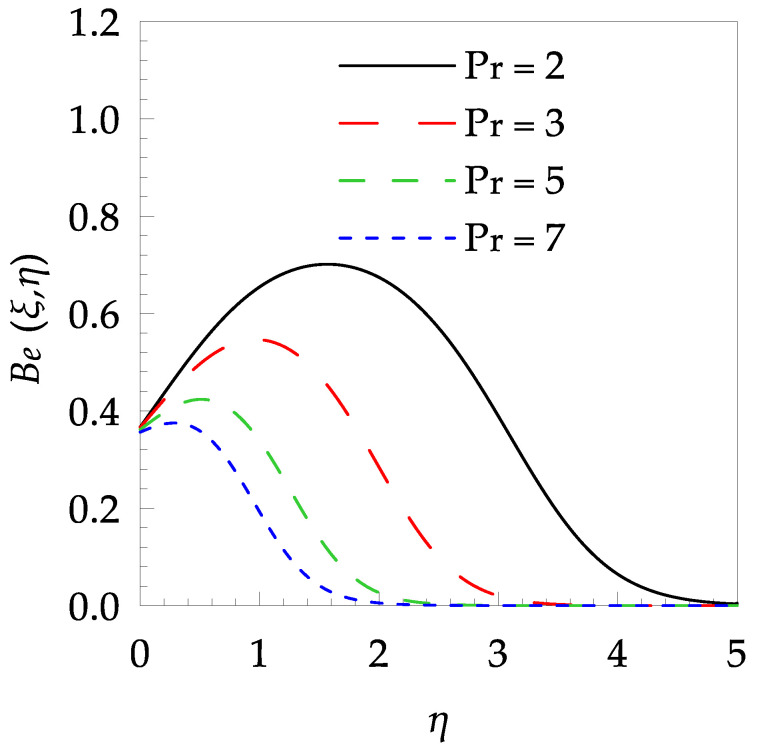
Bejan number profile Be for some values of Pr, when ξ=0.5, Ω=0.8, M=1.5 and Ec=0.2.

**Figure 15:**
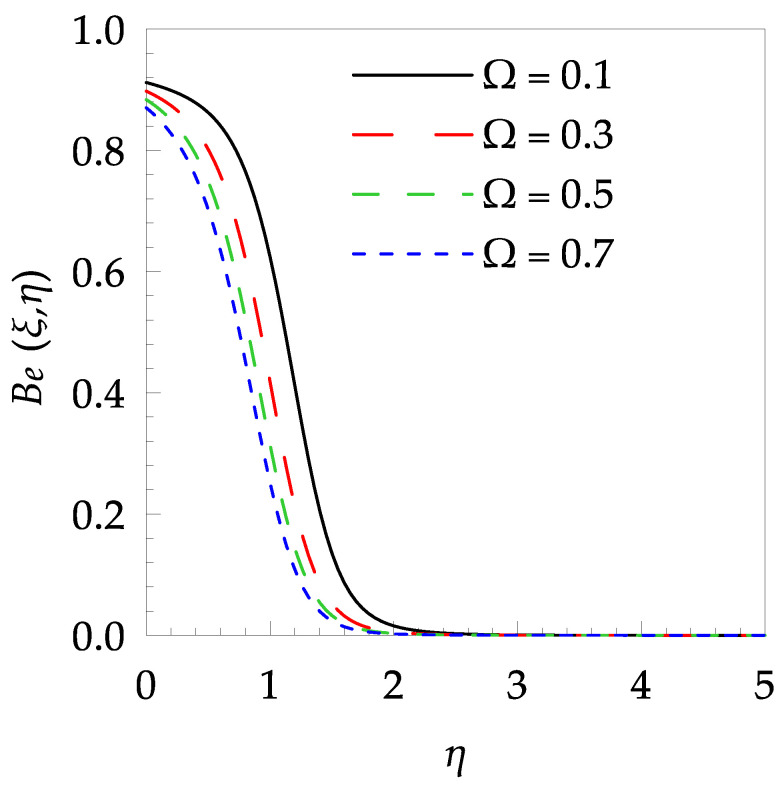
Bejan number profile Be for some values of Ω, when ξ=0.4, Pr=7, M=0.5 and Ec=0.1.

**Figure 16:**
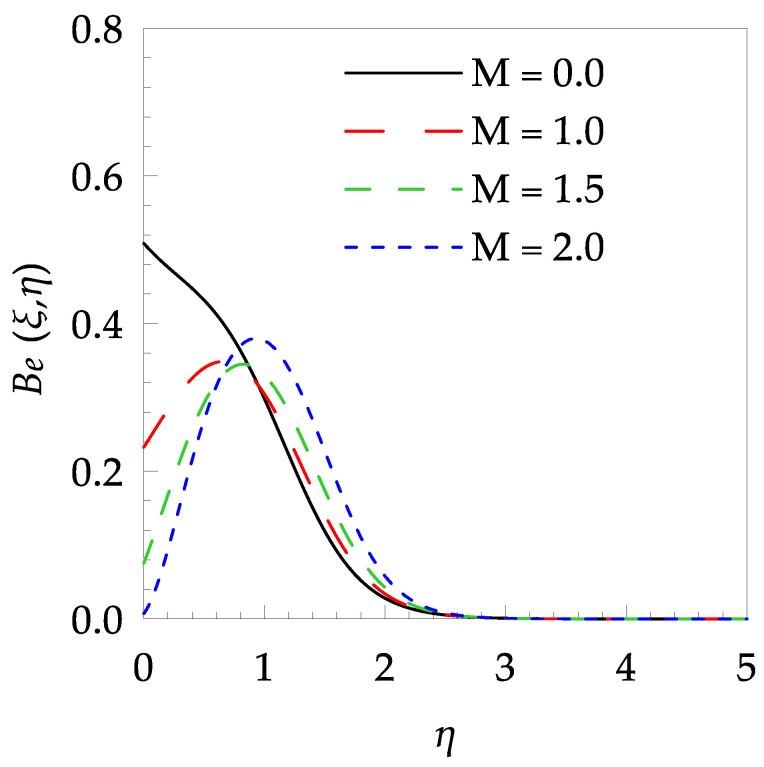
Bejan number profile Be for some values of M, when ξ=0.3, Pr=5, Ω=0.6 and Ec=0.5.

**Figure 17:**
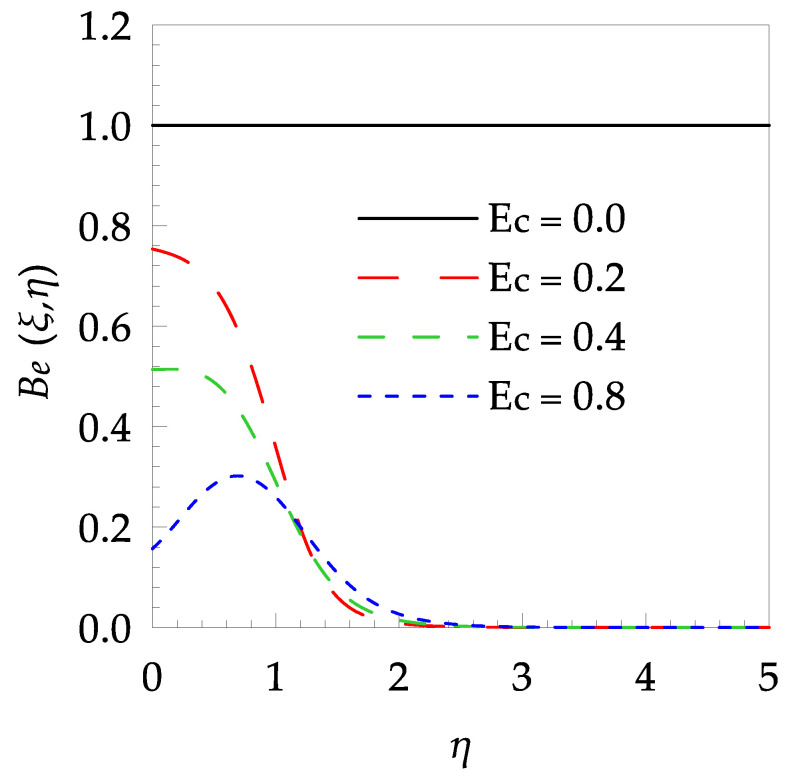
Bejan number profile Be for some values of Ec, when ξ=0.2, Pr=7, Ω=0.4 and M=0.5.

In this paper, the behaviors of velocity and temperature fields, skin friction coefficient, Nusselt number, entropy generation and Bejan number toward the involved pertinent parameters are examined numerically using the Gear-Generalized Differential Quadrature Method (GGDQM). In addition, the present numerical outputs are validated and discussed clearly and wittily via several graphical and tabular illustrations as shown in Figure 2, Figure 3, Figure 4, Figure 5, Figure 6, Figure 7, Figure 8, Figure 9, Figure 10, Figure 11, Figure 12, Figure 13, Figure 14, Figure 15, Figure 16 and Figure 17 and Table 1, Table 2, Table 3, Table 4, Table 5 and Table 6.

Table 6 shows the effects of all physical parameters over the skin friction coefficient Rex1/2Cfx and Nusselt number Rex−1/2Nux for the unsteady flow. It is observed that both Rex1/2Cfx and Rex−1/2Nux decreases as time parameter ξ increases. Skin friction coefficient Rex1/2Cfx increases; however, the Nusselt number Rex−1/2Nux decreases when increasing the Hartman number. Nusselt number Rex−1/2Nux increases for increasing values of Prandtl number, whereas the opposite behavior is seen for rising values of Eckert number. Figure 2 is sketched to see the variation of time parameter ξ. As ξ increases the velocity $f_{\eta }\left(\xi ,\eta \right)$ and momentum boundary layer thickness decrease. The impact of magnetic interaction parameter M on the speed shape is observed in Figure 3. Boundary layer thickness and velocity decline as M increases. Physically, the magnetic field is correlated with the electrically conducting fluid and creates a Lorentz force that is opposite to the direction of fluid flow; consequently, fluid velocity decreases. Figure 4 shows that temperature θ(ξ,η) lessens by increasing ξ. Figure 5 exhibits the impact of Prandtl number on temperature θ(ξ,η). It is clear that temperature dropped with increasing Prandtl number. When increasing the Prandtl number (decreasing the thermal conductivity of fluid), the heat flow rate from the stretching boundary trims down and consequently the thermal boundary layer descends. Growing M decelerates the fluid flow; therefore, the friction between the fluid layers increases and generates frictional heating that raises the temperature (see Figure 6). The effect of Eckert number (Ec) on the temperature is shown in Figure 7. As expected, temperature mounts with mounting values of Ec. By increasing Ec, the resistance sandwiched between the fluid adjoining layers increases and leads to a change of the kinetic energy into thermal energy. Furthermore, as Ec increases, the width of the thermal boundary layer also increases. The influence of ξ on Ns is exposed in Figure 8. At the boundary and near to it entropy increases with ξ. However, after reaching a maximum value a decreasing trend is observed. Figure 9 displays the effects of Pr on entropy production number Ns. It is found that the entropy creation number is a rising function of Pr (due to high-temperature gradients) in the boundary layer flows. Figure 10 specifies the deviation of entropy creation number with dimensionless temperature Ω. It is observed that entropy decreases with increasing values of Ω. Hence, one can attain the main goal, that is, entropy creation minimization by reducing the working temperature disparity $\left(T_{w}-T_{\infty }\right)$. Figure 11 specifies that entropy near the surface increases with M but after a certain η entropy decreases Ns. Figure 12 represents the effect of Ec on entropy production number Ns and increasing behavior is observed. Figure 13 demonstrates the impacts of ξ on Be. The figure shows that Be decreases with an increase in ξ. The impact of Prandtl on Bejan number Be is illustrated in Figure 14. With a large Prandtl number Pr Bejan number decreases. Figure 15 and Figure 16, shows that Be declines when raising the dimensionless temperature difference Ω and Hartmann number M, respectively. Figure 17 portrays the Be for different values of Ec. Note that for Ec=0, entropy creation is only due to heat transport. As the Eckert number is inversely proportional to the temperature difference, for high temperature dissimilarity between the surface and the ambient fluid, the viscous dissipation parameter becomes zero (Ec=0.0). Therefore, the heat transfer irreversibility in the entire flow region is completely dominant, i.e., (Be=1.0).

## 5. Closing Remarks

The impacts of energy dissipation and magnetic field on heat transfer and entropy generation are analyzed by utilizing a new hybrid numerical technique called gear-generalized differential quadrature method (GGDQM). The flow driven by a stretching boundary is assumed to be unsteady. Following are the key findings:

Fluid decelerates with the enhancement of reduced dimensional time and magnetic parameter. 

Increase in reduced dimensional time and Prandtl number reduced the fluid temperature and reverse behavior was observed with rising values of Eckert number and magnetic parameter.

Entropy generation number Ns, rises with enhancing values of reduced dimensionless time at the boundary and its vicinity. The effects become opposite after the certain distance from the stretching boundary. 

Entropy generation number Ns, enhances with magnetic parameter, Prandtl and Eckert number. Reduction in Ns is observed with rising values of Ω. 

The effects of emerging parameters on Ns are significantly prominent at the surface of stretching boundary.

Decrement in Bejan number Be is observed at the surface of stretching sheet with enhancing values of Eckert number, magnetic parameter, Prandtl number and temperature difference parameter. 

## Figures and Tables

**Table 1:** Comparison of our numerical results with those obtained by Agbaje and Motsa [54] for $\theta_{\eta }\left(\xi ,0\right)$ at different values of Pr, when ξ=0.5, Ec=0 and M=1.

Pr	SPM [54]	SRM [54]	GGDQM	
	$\Delta \xi =10^{-4}$	$\Delta \xi =10^{-4}$	$\Delta \xi =10^{-4}$	$\Delta \xi =10^{-4}$
0.7	−0.6278318239	−0.6278318241	−0.6278318240	−0.6278318238
1.5	−0.9704104930	−0.9704104933	−0.9704104926	−0.9704104929
3.0	−1.4270081804	−1.4270081807	−1.4270081793	−1.4270081804
5.0	−1.8845313181	−1.8845313184	−1.8845313162	−1.8845313181
7.0	−2.2577308115	−2.2577308118	−2.2577308091	−2.2577308115
10	−2.7291527800	−2.7291527804	−2.7291527770	−2.7291527800

**Table 2:** Comparison between the exact values of $f_{\eta \eta }\left(\xi ,0\right)$ and those obtained by GGDQM at different values of M, for the limiting cases ξ=0 and ξ=1.

M	$f_{\eta \eta }\left(\xi ,0\right)$			
	ξ=0		ξ=1	
	Exact Results	GGDQM	Exact Results	GGDQM
0.0	−0.56418958	−0.56418958	−1.0000000	−1.0000000
1.0	−0.56418958	−0.56418958	−1.4142135	−1.4142135
1.5	−0.56418958	−0.56418958	−1.8027756	−1.8027756
2.0	−0.56418958	−0.56418958	−2.2360679	−2.2360679

**Table 3:** Comparison between the exact values of $\theta_{\eta \eta }\left(\xi ,0\right)$ and those obtained by GGDQM at different values of Pr, M and Ec, for the limiting cases ξ=0 and ξ=1.

Pr	M	Ec	$\theta_{\eta \eta }\left(\xi ,0\right)$			
			ξ=0		ξ=1	
			Exact Results	GGDQM	Exact Results	GGDQM
4.0	0.0	1.0	−1.2732395	−1.2732395	4.0000000	4.0000000
4.0	1.0	1.0	−1.2732395	−1.2732395	−4.0000000	−3.9999999
4.0	1.5	1.0	−1.2732395	−1.2732395	−14.0000000	−14.0000000
4.0	2.0	1.0	−1.2732395	−1.2732395	−28.0000000	−28.0000000
6.0	0.5	0.0	0.0000000	0.0000000	12.0000000	12.0000000
6.0	0.5	0.5	−0.9549296	−0.9549296	7.5000000	7.4999999
6.0	0.5	1.5	−2.8647889	−2.8647889	−1.5000000	−1.5000001
6.0	0.5	2.0	−3.8197186	−3.8197186	−6.0000000	−6.0000001

**Table 4:** Comparison of our numerical results for $f_{\eta \eta }\left(\xi ,0\right)$ with those obtained by Motsa et al. [53] at different values of ξ and Δξ, when M=0.

ξ	Method	Δξ				
		0.01	0.001	0.0005	0.0002	0.0001
0.1	SRM [53]	−0.61046835	−0.61046762	−0.61046761	−0.61046761	−0.61046761
	SQLM [53]	−0.61045544	−0.61046674	−0.61046742	−0.61046758	−0.61046761
	GGDQM	−0.61041972	−0.61046718	−0.61046751	−0.61046759	−0.61046761
0.3	SRM [53]	−0.70126751	−0.70126681	−0.70126680	−0.70126680	−0.70126680
	SQLM [53]	−0.70126943	−0.70126664	−0.70126676	−0.70126679	−0.70126680
	GGDQM	−0.70125747	−0.70126671	−0.70126678	−0.70126680	−0.70126680
0.5	SRM [53]	−0.78982903	−0.78982837	−0.78982837	−0.78982837	−0.78982837
	SQLM [53]	−0.78981759	−0.78982831	−0.78982835	−0.78982836	−0.78982837
	GGDQM	−0.78982519	−0.78982833	−0.78982836	−0.78982836	−0.78982837
0.7	SRM [53]	−0.87626715	−0.87626654	−0.87626653	−0.87626653	−0.87626653
	SQLM [53]	−0.87625663	−0.87626652	−0.87626653	−0.87626653	−0.87626653
	GGDQM	−0.87626547	−0.87626652	−0.87626653	−0.87626653	−0.87626653
0.9	SRM [53]	−0.96053875	−0.96053800	−0.96053800	−0.96053800	−0.96053800
	SQLM [53]	−0.96053069	−0.96053800	−0.96053800	−0.96053800	−0.96053800
	GGDQM	−0.96053779	−0.96053799	−0.96053799	−0.96053800	−0.96053800

**Table 5:** Comparison of our GGDQM computational times with those obtained by Motsa et al. [53] for $f_{\eta \eta }\left(\xi ,0\right)$ at different values of ξ, when M=0 and $\Delta \xi =10^{-4}$.

ξ	CPU Time (s)		
	SRM [53]	SQLM [53]	GGDQM
0.1	01.93	04.72	5.019
0.3	06.01	14.67	5.326
0.5	10.69	24.19	5.612
0.7	15.08	33.29	5.930
0.9	19.57	42.65	6.320

**Table 6:** Approximate numerical values of Rex1/2Cfx and Rex−1/2Nux computed by GGDQM at different values of *ξ*, Pr, M and Ec.

ξ	Pr	M	Ec	Rex1/2Cfx	Rex−1/2Nux
**0.1**	6.0	0.8	0.7	2.0415507	2.1732328
**0.2**				1.6200792	1.8904622
**0.3**				1.4625827	1.8127995
**0.5**				1.3401177	1.7802112
0.5	**2.0**	1.5	0.2	1.8162426	1.5214377
	**3.0**			1.8162426	1.8609960
	**5.0**			1.8162426	2.3780814
	**7.0**			1.8162426	2.7823358
0.3	5.0	**0.0**	0.5	1.2803321	2.6073736
		**1.0**		1.5602619	2.0405642
		**1.5**		1.8757871	1.3748750
		**2.0**		2.2665979	0.5145979
0.2	7.0	0.5	**0.0**	1.5276915	4.6016634
			**0.2**	1.5276915	3.9378840
			**0.4**	1.5276915	3.2741046
			**0.8**	1.5276915	1.9465459
